# The knowledge attitude and practice (KAP) of mothers of asthmatic children toward asthma in Khartoum asthma clinics

**DOI:** 10.1038/s41598-019-48622-2

**Published:** 2019-08-20

**Authors:** Ahmed Abdulgadir Noureddin, Kamil Mirghani Shaaban, Sagad Omer Obeid Mohamed, Ahmed Ali Abdalla, Ahmed Abdallah Ali Mahmoud, Mohammed S. Tawer Salman

**Affiliations:** 1 Faculty of Medicine, University of Khartoum, Khartoum, 11111 Sudan; 2 The Princess Royal Hospital, Grainger drive, Apley, Telford, TF1 6TF UK

**Keywords:** Public health, Paediatrics

## Abstract

Prevention of asthma attacks is one of the major challenges of public health. Sufficient knowledge and positive attitudes and correct practices are crucial for the prevention of exacerbations. However, there is insufficiency of information in regard to these in Sudan. The aim of this study was to assess the knowledge and to identify the attitude and practice of mothers of asthmatic children concerning their use of inhalers, compliance to preventers and to measure its effect on the severity of the disease in their children. A sample of 100 consecutive mothers of asthmatic children was enrolled. Any mother with a child diagnosed with bronchial asthma for more than 3 months, and who attended the outpatient clinic of paediatric asthma in Soba or Ahmed Gasim hospitals or the Emergency room of Ahmed Gasim or Ibrahim Malik hospital in the period from 1st to 31st of October 2016 was eligible to be included. Asthma was believed to be infectious by 7% of the respondents. 17% of the mothers thought asthma was preventable by a vaccine. 21% found inhaler use unacceptable. Half of the mothers (50%) did not use the inhaler correctly. Most of the mothers (69%) did not use the inhaler if symptoms were mild and 53% didn’t use preventers. The severity of asthma was found to be significantly associated with the attitude and practice of mothers (P < 0.05). In conclusion, sustained efforts are required to increase knowledge about all dimensions of asthma and its management among patients and to disperse myths and misguided judgments regarding the disease and its treatment.

## Introduction

Clinical manifestations of asthma can be controlled with appropriate treatment. There should be only occasional symptoms and no serious asthma attacks^1,2^. This depends on developing a doctor/patient relationship to identify and reduce exposure to risk factors then assess, treat, and monitor asthma and prevent attacks. Hence knowledge about asthma and its risk factors and triggers and the attitude toward the management and the use of inhalers is crucial. Despite the availability of simple and effective therapies, the proportions of children reported to have asthma symptoms is high in Sudan, including 12% of children in Khartoum^3,4^. The objective of this study was to assess the knowledge of the mothers and to identify their attitude and practices toward their childrenssess the knowledge of the mothe to try to understand why asthma treatment is so ineffective.

## Methods

### Study design and setting

This was a descriptive cross-sectional hospital based study done in asthma clinics in three hospitals in Khartoum state (Soba university hospital, Ahmed Gasim teaching hospital and Ibrahim Malik teaching hospital) between the 1st and 31st of October 2016. These settings were chosen because they are major public health, tertiary healthcare facilities providing specialized clinical inpatient and outpatient services for a significant number of the Khartoum state population. A sample of 100 consecutive mothers of asthmatic children was enrolled. Inclusion criteria were that the mothers had a child diagnosed with bronchial asthma at least 3 months previously, who attended the Paediatric outpatient department; those who declined to participate were excluded.

### Data collection and analysis

A structured interview using a questionnaire was carried out, modified from an existing validated tool^5^. We determined the sociodemographic data of the mother and the child and asked questions about knowledge about the etiology of the disease, triggering factors, signs and symptoms. We determined the attitude to the use of inhalers and the practice of the mother toward asthma management and compliance to control plan, and assessment of the severity of child’s asthma. Severity was assessed by 4 question about the recent history of the disease. The questions were about occurrence of the symptoms, the effect on activity, night waking because of asthma and the use of short acting bronchodilators or ER visits based on GINA and national heart, lung and blood institute (NHLBI) guidelines^6,7^. The child was classified into intermittent, mild persistent, moderate persistent, and severe persistent asthma. A scoring system was generated and participants were given a score on each correct answer provided. The collected data was entered and analyzed by statistical package for the social sciences (SSPS) version 23. Chi-square test was used to determine the association between the independent categorical variables and the main outcomes of this study. Significance level was set as p- value of equal to or less than 0.05.

### Disclosure

The abstract of this study was accepted to be presented as poster in the Austrian chest physician society conference in October 2017 and was published in the Central European Journal of Medicine.

Cited: Wien Klin Wochenschr (2017) 129: 743. 10.1007/s00508-017-1273-0.

### Ethics approval and consent to participate

Permission for conducting this research was granted by the institutional review board, University of Khartoum faculty of Medicine prior to study initiation, and from general directors of Ibrahim Malik, Ahmed Gasim and Suba hospitals, Khartoum, Sudan. Ethical approval was obtained from the State Ministry of Health in Khartoum state, Sudan. Each respondent’s provided informed consent prior to participation. And all methods were performed in accordance with the relevant guidelines and regulations.

## Results

### Socio-demographic characteristics of the participants

A total of 100 mothers of 69 males and 31 female asthmatic children were involved in this study. (57%) of the mothers aged from 30 to 40 years. (11%) of them were illiterates and (52%) were secondary school graduates, (63%) of them were housewives. (8%) of the children aged less than 1 year, (44%) aged from 1 to 5 years, (27%) are 5 to 10 years and (23%) were older than 10 years. Regarding the duration of asthma, (38%) of them were diagnosed 3 months to 1 year ago, (44%) diagnosed for 1 to 5 years and (18%) diagnosed for more than 5 years ago. Nearly half of the children (47%) had intermittent asthma, (25%) had mild persistent asthma, (22%) had moderate persistent asthma and (6%) had severe persistent asthma (Table 1).

**Table 1:** Socio-demographic characteristics of the respondents.

Socio-demographic		Percent
Mothers age	20–30 years	21%
	30–40 years	57%
	40–50 years	12%
	>50 years	10%
Mothers Educational level	Illiterate	11%
	Primary school	22%
	Secondary School	52%
	University	15%
Mothers Occupation	House wife	63%
	Employee	28%
	Worker	9%
Duration of asthma	3 month–1 year	38%
	1–5 years	44%
	>5 years	18%
Severity of asthma	Intermittent	47%
	Mild persistent	25%
	Moderate persistent	22%
	Severe persistent	6%

**Figure 1:**
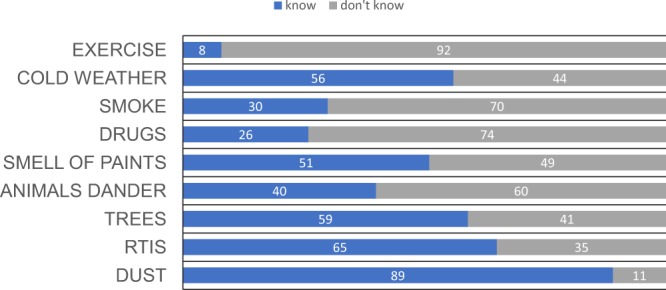
Influencing and triggering factors. RTIs: respiratory tract infections.

38% mothers did not know what caused asthma, but 92% thought it was hereditary. 17% thought that asthma could be prevented by immunization. 30% of the mothers believed that asthma is only an acute disease. 96% of them knew that it affects both children and adults. Although 89% of the mothers knew dust could trigger asthma, but 92% did not know about exercise induced asthma, 70% did not know about smoke triggering asthma and 74% didn’t know about drugs effect in triggering asthma (Fig. 1).

Regarding symptoms of asthma 69% of the mothers did not know that tachycardia is an asthma symptom and 50% did not mention night awakening. Most did know about cough (85%), shortness of breath (85%), and wheeze (73%). 70% knew that asthma can kill if severe. Educational level of the mothers was found to be associated with their level of knowledge significantly p = 0.013.

### Attitude of the mothers

**Figure 2:**
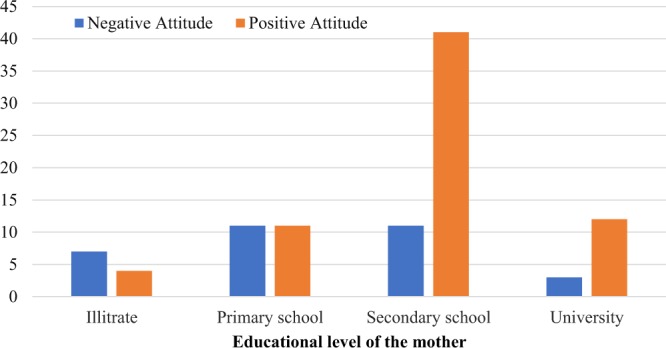
The association the educational level and the attitude of the mothers. P = 0.007.

The study showed that 88% of mothers were anxious about their asthmatic children going outside and having an asthma attack. Although 85% thought taking medications to be necessary to prevent asthma attacks, there were 36% who did not believe that steroid inhalers (preventers) and other prevention treatments can control asthma, and there was 21% that didn’t accept the use of inhalers. There was significant association between mother’s educational level and their attitude p = 0.007 (Fig. 2).

### Practice of the mothers

The mothers were handed an inhaler and were asked to demonstrate how to use it and this was assessed by the investigator. 50% did not know use the inhaler correctly. Mothers were asked if they used the quick relief inhaler for mild symptoms; 69% answered no, 9% said sometimes and 22% answered yes. Regarding long-term steroid inhalers (preventers) use, 53% stated that they did not use them, and of the 47 that did use steroid inhalers, only 25 (53%) used them as prescribed.

The study showed significant associations between the attitude of the mothers and severity of their children’s asthma (p = 0.021), and between the practice of the mothers and the severity of the disease (P = 0.003). There were also significant association between asthma severity and the correct use of inhalers (P = 0.009), with the use of quick relief inhaler in mild symptoms (P = 0.006), and with the application of protection from triggering factors (P = 0.001). (Table 2).

**Table 2:** Association between severity of asthma and KAP.

		Severity of asthma				P value
		Intermittent	Mild persistent	Moderate persistent	Severe persistent	
Knowledge	poor knowledge	9	3	3	1	0.561
	moderate knowledge	27	20	15	3	
	good knowledge	11	2	4	2	
Attitude	negative attitude	15	3	10	4	0.021
	positive attitude	32	22	12	2	
Practice	good practice	17	17	6	0	0.003
	poor practice	30	8	16	6	
Proper using of the inhaler	yes	23	18	9	0	0.009
	no	24	7	13	6	
	yes	14	5	3	0	
Using inhaler in mild symptoms	no	30	14	19	6	0.006
	sometimes	3	6	0	0	
Protection from triggering factors	yes	41	25	13	4	0.001
	no	6	0	9	2	

## Discussion

### Statement of principal findings

This study assessed KAP towards asthma in asthma clinics in three hospitals in Khartoum, Sudan. The study targeted the mothers because they are the ones who take care of the children and they are almost always the co-patient to their sick children. In general, the results of the current study showed an insufficient level of knowledge about asthma and a wide gap between recommended and actual practices. The level of education of the mothers was found to be associated with their level of knowledge. Maybe because higher educated mother has better perception of the disease. The attitude, practice, course of the disease, proper use of inhalers, use of inhalers in mild symptoms and the use of anti-inflammatory inhalers all these factors strongly affected the severity of the disease in the children.

### Strengths and weaknesses of the study

The current study addresses an important issue and contributes to providing some better understanding of the problem. To the best of our knowledge, this is the first study that assessed the KAP level towards asthma among mothers of asthmatic children in Sudan. However, the findings of this study need to be considered in the context of some limitations; this is a cross-sectional study done in three sites which might limit results generalization for all settings in the country, and inclusion of small sample size could compromise representativeness.

### Strengths and weaknesses in relation to other studies

Similar to the findings of this study, Jing Zhao *et al*. study found that parents’ education level and parents’ KAP scores were protective factors against asthma attacks^8^. That Chinese study was a multicenter study in 29 cities included 2960 patients and investigated the clinical status of asthma control and severity of asthma in children.

Successful asthma management relies upon many elements, namely adequate education and positive attitude. Parent education is one of the most important components of childhood asthma treatment. Since health-related behaviors are significantly affected by different aspects of KAP^9^, education should not be limited to providing information, but should be aimed at altering behavior and practice.

Successful KAP education programs for parents of asthmatic children have been established in some countries^10,11^. Tyra Bryant-Stephens program showed a successful improvement in asthma knowledge, ability to control child’s asthma, and asthma quality of life^10^. Another population-based asthma management program on pediatric asthma patients and their caregivers by Archelle Georgiou *et al*.^11^ was associated with statistically significant outcomes in reduction in adverse utilization, symptoms, and restricted activity days for children. This program included various staggered educational mailings, reminder aids, videos, and telephonic case management. Randall Brown review suggested eleven elements to be included for any complete asthma education program as needed by learner, and other steps for clinicians to encourage better self-regulation in patients^12^. Also, school-based asthma education programs could raise the awareness of asthma and decrease its impact. A systematic review showed that these programs promote the improvement of knowledge and showed a reduction of the asthma symptoms, hospitalization instances and emergency visits, school absenteeism, and increase in the quality of life of the individuals^13^.

In our study, half of the mothers in the study didn’t know the right way to use the inhalers which is a major problem in controlling asthma in developing countries as 71.8% of Indian patients were using inhaler incorrectly^14^. We found that 21% of the mother didn’t accept the use of inhalers, in another Sudanese study on well-educated adult asthma patients, 12% didn’t accept inhaler use either and they either had no particular clarification or feared deep rooted dependence on inhalers^15^.

### Meaning of the study

We observed a low level of KAP towards asthma among the participants in our study, and asthma severity is significantly affected by different aspects of KAP. The low level of KAP may be attributed to several factors in our setting. Regarding knowledge, there is no information about the non-communicable diseases like asthma in the curriculum of our primary schools or secondary schools and the only source of information to the patients or their parents is their doctors. Unfortunately, most of our patients who have asthma attack come to the emergency room (ER) to take their emergency treatment as nebulized salbutamol and go home feeling well and don’t see a specialist thinking that it is not important. Therefore, they go home without counseling or proper assessment of their condition or prescribed medication.

Regarding practice, most of the mothers have not been taught or trained how to use inhalers and spacer correctly. Most of them were not given guidelines on how to use the medications and control the disease. Proper counseling can increase significantly the knowledge, attitude and practice toward asthma^16^. A Sudanese study conducted in four large district hospitals assessed the data completeness and integrity of filling out of asthma treatment cards. That study had identified important shortcomings, which could affect management of asthma patients^17^.

### Unanswered questions and future research

This study assessed KAP towards asthma among mothers of asthmatic children. However, there is a need for more comprehensive analysis to investigate the possible risk factors that influence KAP level towards asthma and asthma severity in our setting. Understanding of these influencing factors could help us to implement an effective management plan of asthma. Establishing KAP education programs for parents of asthmatic children in our setting is highly recommended.

## Data Availability

The datasets used during the current study are available from the corresponding author on reasonable request.
